# Synthesis, Anticonvulsant and Antinociceptive Activity of New Hybrid Compounds: Derivatives of 3-(3-Methylthiophen-2-yl)-pyrrolidine-2,5-dione

**DOI:** 10.3390/ijms21165750

**Published:** 2020-08-11

**Authors:** Małgorzata Góra, Anna Czopek, Anna Rapacz, Anna Dziubina, Monika Głuch-Lutwin, Barbara Mordyl, Jolanta Obniska

**Affiliations:** 1 Department of Medicinal Chemistry, Faculty of Pharmacy, Jagiellonian University Medical College, Medyczna 9 St., 30-688 Krakow, Poland; malgorzata.gora@doctoral.uj.edu.pl (M.G.); mfobnisk@cyf-kr.edu.pl (J.O.); 2 Department of Pharmacodynamics, Faculty of Pharmacy, Jagiellonian University Medical College, Medyczna 9 St., 30-688 Krakow, Poland; anna.dziubina@uj.edu.pl; 3 Department of Pharmaceutical Biochemistry, Faculty of Pharmacy, Jagiellonian University Medical College, Medyczna 9 St., 30-688, Krakow, Poland; monika.gluch-lutwin@uj.edu.pl (M.G.-L.); barbara.mordyl@uj.edu.pl (B.M.)

**Keywords:** anticonvulsant, antinociceptive, pirrolidyne-2,5-dione, thiophene

## Abstract

The present study aimed to design and synthesize a new series of hybrid compounds with pyrrolidine-2,5-dione and thiophene rings in the structure as potential anticonvulsant and antinociceptive agents. For this purpose, we obtained a series of new compounds and evaluated their anticonvulsant activity in animal models of epilepsy (maximal electroshock (MES), psychomotor (6 Hz), and subcutaneous pentylenetetrazole (*sc*PTZ) seizure tests). To determine the mechanism of action of the most active anticonvulsant compounds (**3**, **4**, **6**, **9**), their influence on the voltage-gated sodium and calcium channels as well as GABA transporter (GAT) was assessed. The most promising compound 3-(3-methylthiophen-2-yl)-1-(3-morpholinopropyl)pyrrolidine-2,5-dione hydrochloride (**4**) showed higher ED_50_ value than those of the reference drugs: valproic acid (VPA) and ethosuximide (ETX) (62.14 mg/kg vs. 252.7 mg/kg (VPA) in the MES test, and 75.59 mg/kg vs. 130.6 mg/kg (VPA) and 221.7 mg/kg (ETX) in the 6 Hz test, respectively). Moreover, in vitro studies of compound **4** showed moderate but balanced inhibition of the neuronal voltage-sensitive sodium (site 2) and L-type calcium channels. Additionally, the antinociceptive activity of the most active compounds (**3**, **4**, **6**, **9**) was also evaluated in the hot plate test and writhing tests, and their hepatotoxic properties in HepG2 cells were also investigated. To determine the possible mechanism of the analgesic effect of compounds **3**, **6**, and **9**, the affinity for the TRPV1 receptor was investigated.

## 1. Introduction

Epilepsy is a chronic disease of the brain that affects around 50 million people worldwide. According to the International League Against Epilepsy, the current classification of seizure types distinguishes three major groups as follows: generalized-onset seizures (with retained or impaired awareness), focal-onset seizures, and unknown-onset seizures [1]. People with epilepsy have usually more psychological problems such as anxiety or depression and injuries such as fractures and bruising related to seizures. Similarly, the risk of early death in people with epilepsy is three times higher than that in the general population, with the highest index of premature death in low- and middle-income countries and in rural areas. Seizures can be controlled, and up to 70% of people with epilepsy live without seizures with the appropriate use of antiseizure drugs. In the remaining 30% of patients, seizures are not sufficiently controlled; hence, new antiepileptic drugs are still being researched [2].

Taking into consideration the complex pathogenesis of epilepsy and difficulties in explaining the mechanism of action of antiepileptic drugs (AEDs), to obtain a new anticonvulsant molecule, we use the modern drug design method named multifunctional ligands (molecular hybridization, multi-target-directed ligands (MTDLs)). Molecular hybridization is an approach in rational drug design where new chemical structures are obtained by combining two or more pharmacophoric units or whole active compounds into a single molecule, which enables to obtain better efficacy and lower side effects than those of the parent drugs. This method has been used with success by many researchers worldwide and has shown very promising results, especially for multifactor or complex diseases such as Alzheimer’s disease, Parkinson’s disease, cancer, inflammation, hypertension, and epilepsy [3].

Pharmacotherapy is the basic method of epilepsy treatment. Currently, we have many antiepileptic drugs (AEDs) with various structures and mechanisms of action. One of them, namely tiagabine, belongs to the third generation of antiepileptic drugs; it is a selective inhibitor of gamma-aminobutyric acid (GABA) reuptake because of its interaction with GABA transporter type 1 (GAT-1). The tiagabine structure contains a bis(3-methylthiophen-2-yl) methane fragment that has been proven to be necessary for blood–brain barrier penetration and for anticonvulsant activity [4,5]. A pharmacophoric fragment with thiophene ring is also present in the structures of other anticonvulsant active compounds, which significantly reduced the number of seizures in mice and showed activity in various animal models of epilepsy [6,7,8]. Beyond anticonvulsant activity, tiagabine exhibited antinociceptive, anxiolytic, and antidepressant potency [9].

Another antiepileptic drug ethosuximide, pirrolidine-2,5-dione derivative, acts by blocking T-type calcium channels and it is used in absence seizure epilepsy. The pyrrolidine-2,5-dione ring is considered as a pharmacophore for many active compounds in the central nervous system, but it is most explored in terms of its anticonvulsant activity [10].

**Figure 1:**
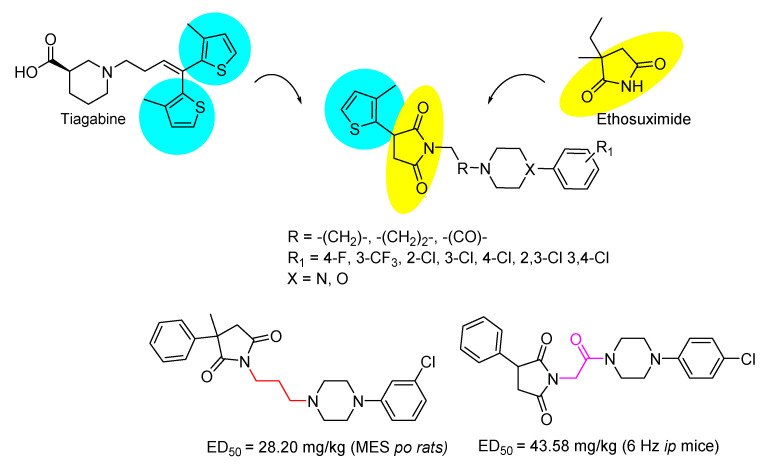
Proposed structural modifications and active compounds obtained in the previous studies [14,15].

In our previous study, we synthesized many derivatives of pyrrolidine-2,5-dione core containing various aliphatic and aromatic substituents at position-3 and an alkylamine(aryl) fragment at position-1 [11,12]. Many of these derivatives showed anticonvulsant activity and were active in the maximal electroshock (MES) test, the subcutaneous pentylenetetrazole (*sc*PTZ) test, and the 6-Hz test. These compounds also showed blocking of sodium and/or calcium ion channels in in vitro studies, which are the major targets of most AEDs [13,14]. In the present study, we designed a new series of hybrid compounds in which 3-methylthiophene ring, which is present in the structure of tiagabine, was combined with pyrrolidine-2,5-dione core present in the well-known antiepileptic drug ethosuximide. In this series, pyrrolidine-2,5-dione ring is linked by two or three methylene carbon linkers with morpholine or 4-arylpiperazine fragment, which contains electron withdrawing fluorine and chlorine atoms or trifluoromethyl group. These 4-arylpiperazine fragments with electron withdrawing atoms or groups were chosen on the basis of structures of the most active pyrrolidine-2,5-dione derivatives described previously [13,14,15]. Moreover, analogues of the aforementioned compounds, in which the methylene carbon linker was replaced with an acetamide fragment, were designed. The structure–activity relationship (SAR) analysis of the previously obtained active pyrrolidine-2,5-dione derivatives revealed that the acetamide moiety may extend anticonvulsant activity in both MES and *sc*PTZ tests [11,12]. The general structure of the newly designed hybrid molecules is shown in Figure 1.

Considering the abovementioned facts with the aim to obtain potent compounds with a broad spectrum of anticonvulsant activity, we synthesized a series of hybrid anticonvulsants containing two pharmacophoric fragments, namely pyrrolidine-2,5-dione and thiophene rings, to ensure that the compound has both mechanisms of action. The newly synthesized hybrid compounds were initially tested in the MES test (model of generalized tonic-clonic seizures) and the 6 Hz psychomotor seizure test (model of focal seizures), and the selected ones that were active in previous tests were assessed in the *sc*PTZ test (model of myoclonic and absence seizures) [16,17,18]. Moreover, for the most active hybrid molecules, the effect of blocking sodium and calcium channels as well as inhibition of GABA reuptake was measured to explain the possible mechanism of action. Bearing in mind that some antiepileptic drugs have been used successfully in other conditions such as neuropathic pain, we also decided to estimate the antinociceptive activity for the most anticonvulsant active compounds by using writhing and hot plate tests and assessed their affinity for TRPV1 receptor.

## 2. Results

### 2.1. Chemical Synthesis and Drug Likeness Properties

**Scheme 1:**
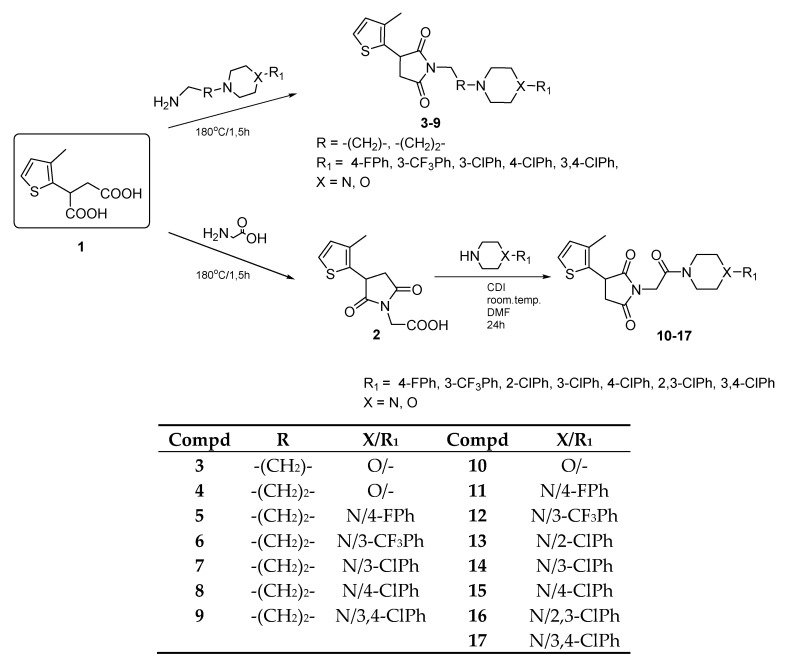
Synthetic route of the target compounds 3–17.

As shown in Scheme 1 the final compounds were obtained in parallel synthetic pathways. Compounds **3**–**9** were synthesized according to the first pathway, in which the starting material 2-(3-methylthiophen-2-yl)succinic acid (1), prepared by the method described by Abeijon et al. [19] was condensed with appropriate aminoalkylmorpholine or 1-(3-aminopropyl)-4-phenylpiperazine. 1-(3-Aminopropyl)-4-phenylpiperazine was obtained by the method described elsewhere [20]. Compounds **10**–**17** were obtained in the second pathway, in which the starting material 2-(3-methylthiophen-2-yl)succinic acid (1) was combined with aminoacetic acid to yield 2-(3-(3-methylthiophen-2-yl)-2,5-dioxopyrrolidin-1-yl)acetic acid (2). This intermediate (2) was converted into final compounds **10**–**17** by coupling with equimolar amounts of appropriate 4-phenylpiperazines or morpholine in the presence of carbonyldiimidazole (CDI). The reaction was carried out at room temperature in dry dimethylformamide for 24 h. The crude products were purified by column chromatography using dichloromethane:methanol mixtures as the development system. Final compounds **3**–**17** as racemic mixtures were obtained with yield ranging between 31% and 86%. Compounds **3**–**9** were obtained as hydrochloride salts, while **10**–**17** were obtained as free bases. Their purity and homogeneity were assessed by TLC and gradient HPLC. The structures of compounds were confirmed by spectral analysis (^1^H-NMR, ^13^C-NMR, LC/MS). The detailed physical and analytical data are listed in the experimental section.

In the next step, using the SwissAdme website [21] physicochemical properties of the final compounds were determined based on Lipinski and Veber rules (Table 1).

The Lipinski and Veber rules are used to evaluate drug likeness or to determine if compounds possess chemical and physical properties to be an orally active drug in humans. Compounds that do not meet at least two of the criteria of the Lipinski rules may cause absorption or permeability problems. The criteria of Lipinski rules are molecular weight (MW) ≤ 500 Da, lipholicity values (log P) ≤ 5, number of hydrogen bond donors (NHD) ≤ 5, number of hydrogen bond acceptors (NHA) ≤ 10, and Veber rules are rotatable bonds (NBR) ≤ 10 and polar surface area (PSA) ≤ 140Ǻ^2^ [22,23].

All tested compounds comply with Lipinski’s rule of five, and contained less than five hydrogen bond donors, less than 10 hydrogen bond acceptors, molecular weights below 500 Da, and logP values < 5. Moreover, in line with the Veber rule, these molecules had the number of rotating bonds (NBR) less than 10 and polar surface area (PSA) values lower than 140 Å^2^.

### 2.2. Anticonvulsant Activity

The profile of anticonvulsant activity of all final compounds was established initially in the MES and psychomotor seizure (6 Hz) tests at the dose of 100 mg/kg, 0.5 h, after intraperitoneal (*ip*) injection into groups of mice, with each group consisting of four animals. Compounds **10**–**17** were evaluated as free bases, and all the other molecules were tested as hydrochloride salts.

The preliminary pharmacological results revealed (Table 2) that compounds **3**, **4**, **6**, **8**, **9**, **10**, **13**, **14**, **15**, and **17** showed anticonvulsant activity in the MES, 6 Hz, or *sc*PTZ tests, whereas compounds **3**, **4**, **6**, **9**, **14**, and **17** showed significant anticonvulsant activity in these tests by protecting minimum two of four tested mice from seizures. Meaningful anticonvulsant activity is defined as protection of at least 50% of the tested mice from seizures. The obtained results showed that in the MES test, both compounds **4** and **9**, unlike tiagabine and ethosuximide, which did not show protection in this test [9,24], at the dose of 100 mg/kg exhibited anticonvulsant activity, but compound **4** was more active because it protected 75% of the tested animals in contrast to compound **9**, which protected 50% of the tested mice. In the 6 Hz seizure model (32 mA), compounds were visibly more potent than that in the MES test; five compounds, namely **3**, **4**, **6**, **14**, and **17** showed antiseizure activity. The highest activity was shown by compound **3**, which protected four of four (100%) tested animals, but compounds **4** and **6** were only slightly less active and demonstrated at least 75% satisfactory protection in this test.

In the rotarod test (NT), which measures acute neurological toxicity, the majority of compounds were devoid of neurotoxicity, but some of them, e.g., **5**, **6**, and **7** like tiagabine [9], showed significant motor impairment in this test (reported in Table 2).

**Figure 2:**
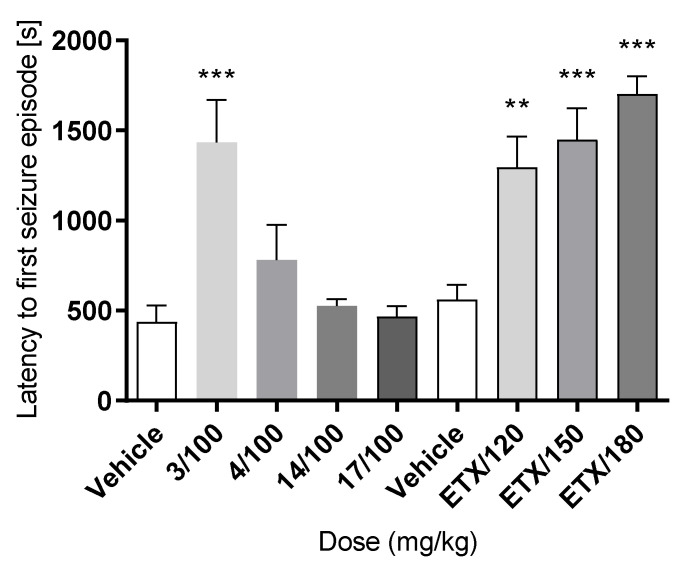
Anticonvulsant activity of compounds **3**, **4**, **14**, **17**, and ethosuximide (ETX) in the *sc*PTZ test. Each value represents the mean ± S.E.M. obtained from 4–6 mice. Statistical analysis: one-way analysis of variance (ANOVA), followed by Dunnett’s post hoc test. Significant difference compared to the control group: ** *p* < 0.01, *** *p* < 0.001. Data for ETX from the previous study [24].

Furthermore, four active compounds in previous tests (**3**, **4**, **14**, **17**) were selected for the evaluation of their activity in the *sc*PTZ test. The results of the *sc*PTZ test showed anticonvulsant activity only for one compound (**3**), which protected 50% of the tested animals. In this test, compound **3** at a dose 100 mg/kg significantly influenced the latency time to first seizure episode. As shown in Figure 2, it prolonged this time from 436.8 ± 91.94 s to 1435 ± 234.9 s (by 228%, *p* < 0.001). Tiagabine was also active in *sc*PTZ test and it prolonged the latency time to first episode, as well as it reduced the number of seizure episodes [9]. On the other hand, no significant effect was observed for compounds **4**, **14**, and **17**.

Based on the preliminary data, for the most active compounds in the initial studies, the median effective doses (ED_50_) were evaluated in the MES and 6 Hz tests, and the median neurotoxic doses (TD_50_) were determined in the rotarod test. These two sets of data were used to calculate protective index (PI), which is a measure of the benefit-risk proportion of the therapeutic agent. Results of the tested compounds with the data for reference AEDs (ethosuximide (ETX) and valproic acid (VPA)) are presented in Table 3. It can be seen that compound **3** exhibited almost two-fold lower ED_50_ value than VPA in the 6 Hz test and similar PI. This compound (**3**) also exhibited three-fold lower ED_50_ value than ETX in the same test. Compound **4** showed more beneficial ED_50_ value in the MES and 6 Hz tests than the two reference drugs used, which was 2–4 times lower than that of ETX and VPA**.** This compound (**4**) also exhibited better PI than VPA in the MES test. Moreover, compound **6** exhibited almost 2–3-fold lower ED_50_ value than ETX and VPA, respectively, in the 6 Hz test. In this test, compound **14** showed ED_50_ value slightly higher than that of VPA.

### 2.3. Antinociceptive Activity

**Figure 3:**
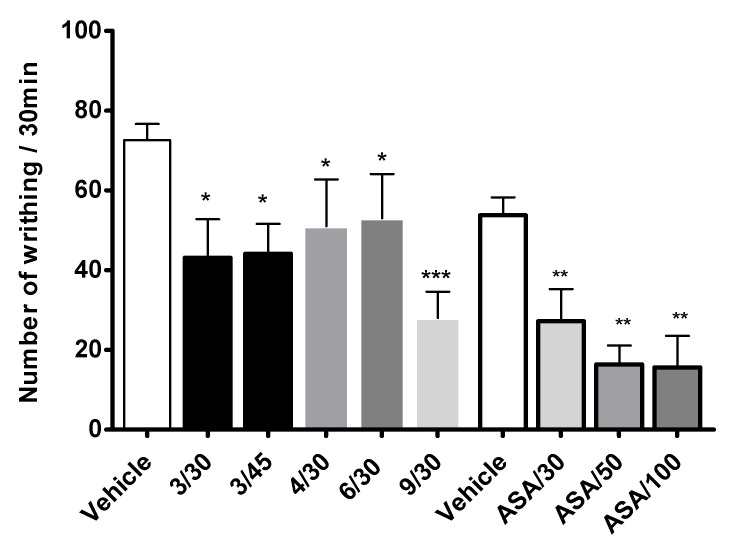
Antinociceptive activity of compounds **3**, **4**, **6**, **9** and acetylsalicylic acid (30, 50, and 100 mg/kg) in the acetic acid-induced writhing test. Data are presented as mean ± SEM and were analyzed by one-way analysis of variance (ANOVA), followed by Dunnett’s post hoc test or *t*-test. Significant difference compared to the vehicle-treated group. * *p* < 0.05, ** *p* < 0.01, *** *p* < 0.001, *n* = 8.

All the tested compounds showed significant analgesic properties (Figure 3), i.e., they reduced acetic acid-induced writhing behavior. The mean number of writhing responses in the vehicle-treated mice was 73. The strongest analgesic effect was observed for compound **9**, which at the dose of 30 mg/kg reduced the number of writhes in response to an irritating stimulus by 61.44%. Moreover, this compound showed analgesic activity comparable to that of aspirin (ASA) (30 mg/kg) used as a reference drug. The administration of compounds **4** and **6** at the same dose produced a weaker analgesic effect (inhibition by 29.8% and 27%, respectively). Compound **3** when applied at the doses of 30 and 45 mg/kg significantly reduced writhing behavior by 34% and 39%, respectively.

**Figure 4:**
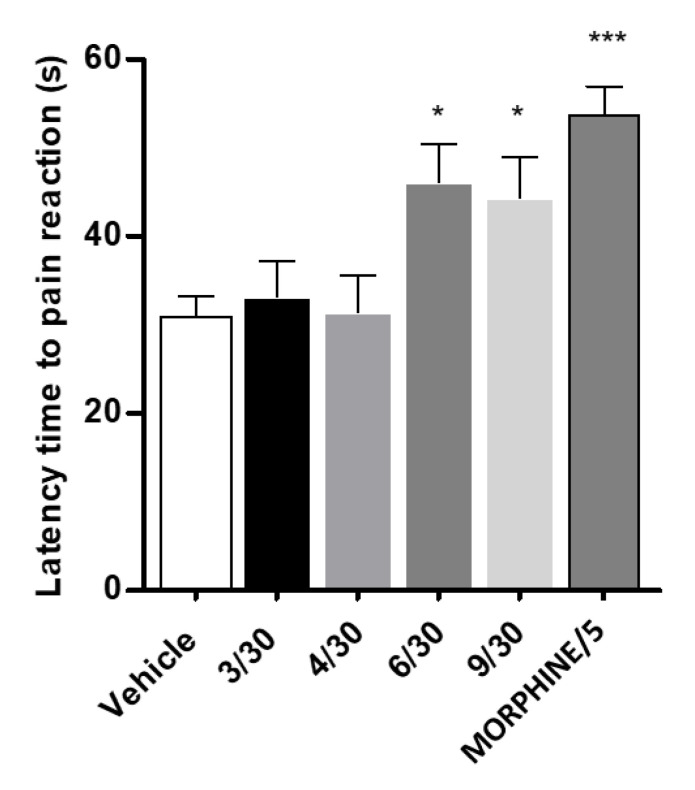
Effects of compounds **3**, **4**, **6**, **9** at the dose of 30 mg/kg, *ip,* and morphine (5 mg/kg) on response latency in the hot plate test in mice. Data are presented as mean ± SEM and were analyzed by one-way analysis of variance, followed by Dunnett’s post hoc test or *t*-test. Significant difference compared to the vehicle-treated group—* *p* < 0.05, *** *p* < 0.001; *n* = 8.

In the hot plate test, of all the tested compounds, only **6** and **9** at the dose of 30 mg/kg significantly prolonged the latency time to pain reaction by 49% (*p* < 0.05) and 43% (*p* < 0.5), respectively (Figure 4). The analgesic activity of tiagabine in this test was observed at a slightly lower dose (8 mg/kg) than the tested compounds **6** and **9** [9]. The other two compounds, i.e., **3** and **4** were found to be inactive in this test.

### 2.4. In Vitro Radioligand Binding Studies

As indicated in Table 4, compound **6** exhibited the highest affinity to the voltage-sensitive Na^+^ channel-site 2 (96.7%), which also revealed a significant effect on voltage-sensitive Ca^2+^ channel L-type (69.9%). Compound **9** also showed high blocking of voltage-sensitive Na^+^ channel-site 2 (78.8%). Compound **4** revealed a moderate and balanced blocking of the voltage-sensitive Ca^2+^ channel L-type (39.7%) and voltage-sensitive Na^+^ channel-site 2 (42.1%). At the same time, the evaluation of GABA-transporter blocking (GAT) was also performed for the most active compounds in the anticonvulsant in vivo tests (**3**, **4**), bearing in mind that the mechanism of action of tiagabine involves blocking of GAT-1. The obtained results revealed that compounds **3** and **4** showed no interaction with the GABA transporter at a concentration of 100 μM.

In the next step, the effect of compounds **3**, **6**, and **9** on the TRPV1 receptor was studied. The obtained results, at the concentration of 100 µM, revealed that compounds **6** and **9** showed 74.8% and 109.5%, respectively, affinity for TRPV1 receptors, while compound **3** exhibited only 14.8% affinity for these receptors.

### 2.5. Hepatotoxicity Study

**Figure 5:**
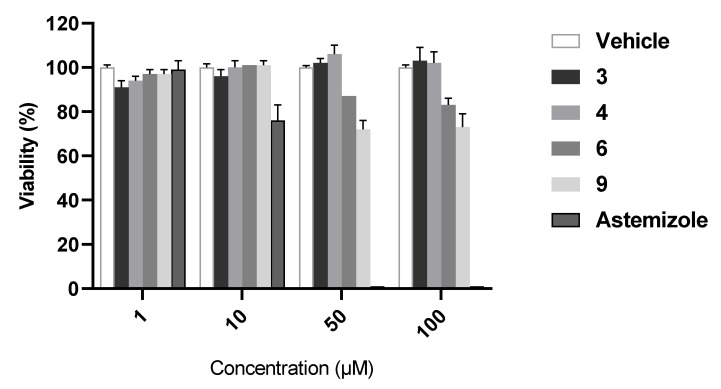
Viability of HepG2 cells incubated in the presence of compounds 3, 4, 6, 9 and astemizole at concentration range 1–100 µM for 24 h. The graph show results from PrestoBlue assay expressed as the percentage of control condition ± SEM. Three independent experiments were performed.

The hepatoxicity results of compounds **3**, **4**, **6,** and **9** showed that two compounds **6** and **9** at the concentrations of 100 and 50 μM inhibited the viability of cells in the range of 13–28%, but at the concentrations of 1 and 10 μM showed no hepatotoxicity. Compounds **3** and **4** revealed no cytotoxic effect on Hep G2 cells in the concentration range of 1–100 μM (cell viability: >94%) (Figure 5). Results are presented in diagram as the percentage of control condition ± SEM.

## 3. Discussion

The results of the present study showed that compounds with the pyrrolidine-2,5-dione core may display interesting biological activity with good bioavailability. Herein we described the anticonvulsant and antinocicepitve activity of fifteen, new 3-(3-methylthiophen-2-yl)pyrrolidine-2,5-dione derivatives. Based on initial results of MES and 6 Hz anticonvulsant tests, few conclusions can be drawn on the structure–activity relationship, in the group of the new hybrid compounds—derivatives of 3-(3-methylthiophen-2-yl)-pyrrolidine-2,5-dione. The anticonvulsant activity depended on the type of linker between pyrrolidine-2,5-dione ring and cyclic amine moiety as well as on the substituent in the 1-phenylpiperazine fragment. Generally, compounds with two or three methylene carbon linkers (**3**–**9**) were more active than those containing acetamide fragment (**10**–**17**). Comparing the two analogues of 3-(3-methylthiophen-2-yl)-pyrrolidine-2,5-dione with methylene carbon linker and morpholine ring (**3**, **4**) higher and more diversified anticonvulsant activity was observed for compound with longer propylene linker (**4**) than for that with ethylene one (**3**). Therefore, for other compounds with methylene carbon linker series (**5**–**9**), a propyl linker was chosen. Among 1-phenylpiperazine derivatives, antiseizure protection was observed for compounds with trifluoromethyl group at position-3 (**6**) and with chlorine atoms at position-3 or -3 and -4 of phenyl ring (**9**, **14**, **17**). Derivatives with two chlorine atoms at position-3 and -4 (**9**, **17**) were active in both series of compounds (with methylene carbon and acetamide linker), but only compound **9**, belonging to the group of derivatives with three methylene carbon linkers, was active in both the MES and 6 Hz tests.

In the next step, for the most promising compounds the median effective doses in maximal electroshock seizures (MES) and in psychomotor seizure (6 Hz) tests were evaluated. The MES test is used to detect compounds that are effective against human generalized tonic–clonic epilepsy, while the 6 Hz test indicates compounds effective against focal type of seizures (32 mA stimulus intensity) and in pharmacoresistant epilepsy (44 mA stimulus intensity). These animal models, particularly MES test, have been routinely used in the early stage of detection of new anticonvulsant compounds in epilepsy research during the last few years. Among them, compound **4** was active in MES test, with ED_50_ value 62.14 mg/kg, while compounds **3**, **4**, **6**, and **14** displayed anticonvulsant activity in 6 Hz test, with ED_50_ values ranging from 74.32 to 153.25 mg/kg. In addition to aforementioned tests, rotarod test showed that majority of the final compounds were devoid of significant neurotoxic effects. Moreover, their permeability through biological membranes, which was initially estimated in silico, showed that all the final compounds are in line with the Lipinski and Veber rules.

Summing up the aforementioned results, compounds **3**, **4**, **6**, and **14** displayed good activity in the 6 Hz test, but among them, compound **4** revealed the broadest spectrum of anticonvulsant activity, since it showed antiseizure properties in MES and 6 Hz tests. It is noteworthy, that the protective index value for compound **4** in MES test was better than the one for the well-known anticonvulsant drug, valproic acid (PI 3.21 vs. 1.7, respectively).

As antiepileptic drugs are used to treat pain of different origins, the pharmacological screening was extended to the evaluation of antinociceptive activity in mice. Four compounds (**3**, **4**, **6**, **9**) were selected for further pharmacological evaluation focused on their antinociceptive activity in acute pain tests in mice. The selection was based on the anticonvulsant activity, diversity of the structures, and neurotoxicity of the chosen compounds. In the present study, the antinociceptive activity of the tested compounds was investigated in two screening models, namely writhing and hot plate tests.

The writhing test is a sensitive method for the preliminary evaluation of antinociceptive activity. In this model, acetic acid directly activates visceral and somatic nociceptors throughout the peritoneum, thereby causing inflammation and tissue damage in the abdominal wall muscles and the visceral organs. Moreover, the administration of dilute acetic acid produces a characteristic writhing response in mice, which is representative of peritoneovisceral inflammatory pain [30]. This test detects peripheral analgesic activity, and antiepileptic drugs with various mechanisms of action also show activity in this test [31,32,33,34,35,36].

The hot plate test is a model of acute, thermally induced pain, and it is a simple and commonly used test for the evaluation of centrally acting analgesics. The mechanism of analgesic effect is considered to be a consequence of supraspinal attenuation of ascending nociceptive input [37,38]. In this test, also antiepileptic drugs with various mechanisms of action (including potential sodium channel blockers) prolong the latency time of pain reaction [14,32,39]. As peripherally acting analgesic drugs are generally inactive in this pain model, morphine was chosen as a reference drug, and it prolonged latency time to pain by 74% (*p* < 0.001).

The strongest antinocicepitve activity was observed for compound **9** in the writhing test at the dose of 30 mg/kg, and it is noteworthy that it was similar to that of aspirin at the same dose. Moreover, two of four of the tested compounds (**6** and **9**) revealed significant peripheral analgesic activity in the hot plate test. However, it is possible that their central analgesic activity in this test may be due to sedative properties. This issue requires further study.

Taking into consideration the fact that the mechanism of action of many antiepileptic drugs is often related to their influence on the activity of sodium and/or calcium channels (e.g., ethosuximide, valproic acid) [40] and interaction with the GABA transporter (e.g., tiagabine), for active compounds in the MES or 6 Hz seizure tests (**3**, **4**, **6**, **9**), the binding evaluation was conducted for sodium (site-2) and L-type calcium channels as well as for GABA transporter [41]. The results of in vitro studies displayed high blockage of neuronal voltage-sensitive sodium (site-2) channels for compounds **6** and **9** (96.7% and 78.8%, respectively), while compound **4** showed moderate effect (42.1%) on these channels. Similarly, the inhibitory effect of compound **6** on L-type calcium channels was quite high (69.9%), whereas it was moderate for compound **4** (39.7%). None of the tested compounds showed interaction with the GABA transporter. It can be concluded that blockage of the neuronal voltage-sensitive sodium (site-2) and L-type calcium channels could be a possible mechanism of action of compounds **4** and **6**.

To extend the in vitro characterization of active compounds in antinociceptive in vivo tests, which could define a possible mechanism of action, we studied the effect of compounds **3**, **6**, and **9** on the TRPV1 receptor. The TRPV1 receptor is the newest and currently an attractive target for anticonvulsants (potential cationic receptor channel, vanilloid ion channel type 1), which is responsible for the sensation of heat and pain (nociception) [42]. Two final compounds, **6** and **9**, showed high and significant affinity for TRPV1 receptors (74.8% and 109.5%, respectively), which may suggest their involvement in analgesic mechanism of action.

As the next step, the hepatoxicity of compounds **3**, **4**, **6**, and **9** was estimated using immortalized hepatocellular carcinoma cells HepG2 in PrestoBlue (Thermo Fisher) Experiment, which measures the metabolic activity of cells. Astemizole, which inhibits the viability of HepG2 cells in a dose-dependent manner, was used as a reference drug. All tested compounds revealed no significant hepatotoxicity at the concentration range 1–100 μM.

As compound **4** demonstrated a broad spectrum of anticonvulsant activity as well as significant peripheral antinociceptive effect, its activity after chronic administration may also be evaluated. Equally noteworthy is compound **9** with its strong central and peripheral analgesic activity. Its precise mechanism of action could be further investigated.

## 4. Materials and Methods

### 4.1. Chemistry

#### 4.1.1. General Remarks

All chemical reagents and solvents were purchased from Sigma–Aldrich (Steinheim, Germany). Melting points (mp.) were measured in open capillaries on a Büchi 353 melting point apparatus (Büchi Labortechnik, Flawil, Switzerland). The purity and homogeneity of the obtained compounds were assessed by thin-layer chromatography (TLC) and high performance liquid chromatography (HPLC). The TLC was performed on Merck silica gel 60 F254 aluminum sheets (Merck; Darmstadt, Germany) using the following developing solvents: S_1_—dichloromethane:methanol (9:0.5, *v*/*v*), S_2_—dichloromethane:methanol (9:0.7, *v*/*v*), S_3_—dichloromethane:methanol (9:1, *v*/*v*). Spots were detected by their absorption under UV light (λ = 254 nm). HPLC analyses were conducted on a Waters HPLC instrument with photodiode array detector Waters 2998 (Waters, Milford, MA, USA), equipped with a Chromolith RP-18 SpeedROD column (4.6 × 50 mm). Conditions applied were a flow rate of 5 mL/min, eluent A (water/0.1% TFA), eluent B (acetonitrile/0.1% TFA); flow rate of 5 mL/min, gradient of 0–100% B over 3 min were used, standard solutions (1 mg/mL) of each compound were prepared in acetonitrile/water (50/50, *v*/*v*), containing a 0.1% TFA, detection at 214 nm. The purity of the investigated compounds 3–19 was > 95%. The UPLC analysis and mass spectra (LC-MS) were obtained on Waters ACQUITY™ TQD system with the TQ Detector (Waters, Milford, MA, USA). The ACQUITY UPLC BEH C18, 1.7 µm, 2.1 × 100 mm column was used (Waters, Milford, MA, USA). NMR spectra were obtained in a FT-NMR 500 MHz JEOL (JNM-ECZR500 RS1 ECZR) apparatus operating at 500 MHz, using a solvent as an internal standard. Chemical shifts are reported in δ values (ppm) and the *J* values are expressed in Hertz (Hz). Signal multiplicities are represented by the following abbreviations: s (singlet), brs (broad singlet), d (doublet), t (triplet), m (multiplet).

#### 4.1.2. Synthesis and Structural Characterization

##### General Procedure for the Synthesis of 2-(3-methylthiophen-2-yl)succinic acid (1) and 2-(3-(3-methylthiophen-2-yl)-2,5-dioxopyrrolidin-1-yl)acetic acid (2)

Starting (*R,S*)-2-(3-methylthiophen-2-yl)succinic acid (**1**) was obtained by using a procedure described elsewhere [19], whereas 2-(3-(3-methylthiophen-2-yl)-2,5-dioxopyrrolidin-1-yl)acetic acid (2) was prepared as follows: 2-(3-methylthiophen-2-yl)succinic acid (0.007 mol) was dissolved in 20 mL of water and 2-aminoacetic acid (0.007 mol) was gradually added. Then, the mixture was heated in a term-regulated sand bath with simultaneous distillation of water. After complete removal of water, the temperature of the reaction mixture was raised up to 180 °C and maintained for ca. 1.5 h. The crude product, 2-(3-(3-methylthiophen-2-yl)-2,5-dioxopyrrolidin-1-yl)acetic acid (**2**) was purified by column chromatography (S_3_: dichloromethane:methanol, 9:1, *v*/*v*) giving the yellow oil.

##### (*R,S*)-2-(3-(3-methylthiophen-2-yl)-2,5-dioxopyrrolidin-1-yl)acetic acid (2)

Yellow oil. Yield: 70.0%; Rf = 0.71 (S_3_); UPLC: *t*_R_ = 4.08; ^1^H NMR (500 MHz, CDCl_3_): δ ppm 2.25 (s, 3H, CH_3_) 2.90 (dd, 1H, CH-pyrrolidyno-2,5-dione, *J*=18.5, 5.6 Hz) 3.33 (dd, 1H, CH-pyrrolidyno-2,5-dione, *J* = 18.5, 9.6 Hz) 4.36 (d, 2H, CH_2_, *J* = 2.4 Hz) 4.43 (dd, 1H, CH-pyrrolidyno-2,5-dione, *J* = 9.6, 5.6 Hz) 6.84 (d, 1H, thiophene, *J* = 5.1 Hz) 7.14 (d, 1H, thiophene, *J* = 5.1 Hz) 7.25 (s, 1H, COOH); ^13^C NMR (126 MHz, CDCl_3_): δ ppm 14.20, 37.63, 39.53, 39.93, 123.43, 130.81, 131.54, 136.43, 170.64, 174.57, 175.92; C_11_H_11_NO_4_S (253.27); Monoisotopic Mass 253.04; [M − H]^+^ = 252.01

##### General Procedure for the Preparation of 1-(3-aminopropyl)-4-arylpiperazines

2-[4-(3-Trifluoromethylphenyl)piperazin-1-yl]propan-1-amine and 3-[4-(3,4-dichlorophenyl)piperazin-1-yl]propan-1-amine were obtained by using a procedure described elsewhere [20]. Additionally, 3-[4-(4-fluorophenyl)piperazin-1-yl]propan-1-amine [43], 3-[4-(3-chlorophenyl)piperazin-1-yl]propan-1-amine [44], 3-[4-(2-chlorophenyl)piperazin-1-yl]propan-1-amine [44], and 3-[4-(4-chlorophenyl)piperazin-1-yl]propan-1-amine [44], as well as 3-[4-(2,3-dichlorophenyl)piperazin-1-yl]propan-1-amine [45]. Additionally, 2-morpholinoethan-1-amine and 3-morpholinopropan-1-amine were purchased from Sigma–Aldrich (Steinheim, Germany).

##### General Procedure for the Synthesis of Compounds 3–9

(*R,S*)-2-(3-Methylthiophen-2-yl)succinic acid (**1**) (0.002 mol) was dissolved in water (10 mL) and substituted 1-aminopropyl-4-arylpiperazine (0.002 mol) or appropriate aminoalkylmorpholine (2-morpholinoethylamine or 3-morpholinopropylamine, 0.002 mol) was added. The mixture was heated up to 100 °C in a temperature-regulated sand bath with simultaneous distillation of water. After complete removal of water, the temperature of the mixture was increased to 180 °C, and this temperature was maintained for 1.5 h. The crude product was purified by column chromatography (dichloromethane:methanol, 9:0.7, *v*/*v*) giving racemic mixture of the final compounds in yields ranging from 54% to 86%. Compounds **3**–**9** were converted into hydrochloride salts according to procedure described elsewhere [15]. In the descriptions below, for compounds **3**-**9**, retention factors (TLC), yield, and LC MS analysis are given for the free base, but NMR spectra and melting points are given for the hydrochloride salts.

##### (*R,S*)-3-(3-Methylthiophen-2-yl)-1-(2-morpholinoethyl)pyrrolidine-2,5-dione hydrochloride (3)

White powdery crystals. Yield: 70.9%; mp. 202.0–204.0 °C; TLC: R_f_ = 0.65 (S_2_); UPLC: *t*_R_ = 2.93 min; ^1^H NMR (500 MHz, CDCl_3_): δ ppm 2.26 (s, 3H, CH_3_) 2.83 (dd, *J* = 18.7, 5.3 Hz, 1H, CH-pyrrolidyno-2,5-dione) 3.19–3.38 (m, 5H, morpholine, CH-pyrrolidyno-2,5-dione) 3.48 (m, 1H, CH-pyrrolidyno-2,5-dione) 3.54 (t, 2H, CH_2_, *J* = 6.2 Hz) 3.72–3.84 (m, 2H, morpholine) 4.18 (t, 2H, CH_2_, *J* = 6.5 Hz) 4.35–4.49 (m, 2H, morpholine) 6.85 (d, 1H, thiophene, *J* = 4.7 Hz) 7.13 (d, 1H, thiophene, *J* = 5.3 Hz); ^13^C NMR (126 MHz, CDCl_3_): δ ppm 14.08, 32.51, 38.15, 40.36, 52.26, 54.75, 63.46, 123.01, 130.73, 131.93, 136.70, 176.30, 178.10; LC/MS for base: C_15_H_20_N_2_O_3_S (308.40); Monoisotopic Mass 308.12; [M + H]^+^ = 309.16.

##### (*R,S*)-3-(3-Methylthiophen-2-yl)-1-(3-morpholinopropyl)pyrrolidine-2,5-dione hydrochloride (4)

White powdery crystals. Yield: 86.2%; mp. 211.0–213.0 °C; TLC: R_f_ = 0.44 (S_2_); UPLC: *t*_R_ = 3.15 min; ^1^H NMR (500 MHz, CDCl_3_): δ ppm 2.17–2.22 (m, 2H, CH_2_) 2.23 (s, 3H, CH_3_) 2.71–2.87 (m, 3H, morpholine, CH-pyrrolidyno-2,5-dione), 3.00–3.08 (m, 2H, morpholine) 3.31 (dd, 1H, CH-pyrrolidyno-2,5-dione, *J* = 18.4, 9.5 Hz) 3.40–3.50 (m, 2H, CH_2_) 3.65 (t, 2H, CH_2,_ *J* = 6.3 Hz) 3.94 (br. s., 2H, morpholine) 4.16 (br. s., 2H, morpholine) 4.46 (dd, 1H, CH-pyrrolidyno-2,5-dione, *J* = 9.5, 5.0 Hz,) 6.82 (d, 1H, thiophene, *J* = 5.1 Hz) 7.10 (d, 1H, thiophene, *J* = 5.2 Hz); ^13^C NMR (126 MHz, CDCl_3_): δ ppm 14.17, 22.41, 36.06, 37.74, 39.88, 51.97, 55.30, 63.69, 123.07, 130.91, 132.08, 136.57, 175.82, 177.31; LC/MS for base: C_16_H_22_N_2_O_3_S (322.42); Monoisotopic Mass 322.14; [M + H]^+^ = 323.18.

##### (*R,S*)-1-(3-(4-(4-Fluorophenyl)piperazin-1-yl)propyl)-3-(3-methylthiophen-2-yl)pyrrolidine-2,5-dione hydrochloride (5)

White powdery crystals. Yield: 54%; mp.160.0–162.0 °C; TLC: R_f_ = 0.59 (S_2_); UPLC: *t*_R_ = 4.54 min; ^1^H NMR (500 MHz, DMSO-*d*_6_): δ ppm 1.94–2.01 (m, 2H, CH_2_) 2.12–2.15 (m, 3H, CH_3_) 2.65 (dd, 1H, CH-pyrrolidyno-2,5-dione, *J* = 17.9, 5.5 Hz) 3.03–3.14 (m, 6H, piperazine, CH_2_) 3.26 (dd, 1H, CH-pyrrolidyno-2,5-dione, *J* = 17.9, 9.4 Hz) 3.43–3.51 (m, 4H, piperazine) 3.67 (d, 2H, CH_2_, *J* = 9.3 Hz) 4.56 (dd, CH-pyrrolidyno-2,5-dione, *J* = 9.4, 5.5 Hz), 6.85 (d, 1H, thiophene, *J* = 5.2 Hz) 6.94–7.01 (m, 2H, ArH) 7.02–7.09 (m, 2H, ArH) 7.31 (d, 1H, thiophene, *J* = 5.2 Hz); ^13^C NMR (126 MHz, DMSO-*d*_6_): δ ppm 14.25, 22.37, 36.22, 37.84, 46.65, 51.08, 53.40, 116.05 (d, *J* = 21.73 Hz), 118.45 (d, *J* = 7.85 Hz), 123.88, 131.00, 133.27, 136.13, 146.82 (d, *J* = 1.81 Hz), 157.20 (d, *J* = 237.21 Hz), 176.14, 177.59; LC/MS for base: C_22_H_26_FN_3_O_2_S (415.53); Monoisotopic Mass 415.17; [M + H]^+^ = 416.30.

##### (*R,S*)-3-(3-Methylthiophen-2-yl)-1-(3-(4-(3-(trifluoromethyl)phenyl)piperazin-1-yl)propyl)pyrrolidine-2,5-dione hydrochloride (6)

White powdery crystals. Yield: 59.5%; mp. 183.0–185.0 °C; TLC: R_f_ = 0.60 (S_2_); UPLC: *t*_R_ = 5.25 min; ^1^H NMR (500 MHz, CDCl_3_): δ ppm 1.83 (quin, 2H, CH_2_, *J* = 7.1 Hz) 2.24 (s, 3H, CH_3_) 2.43 (t, 2H, CH_2_, *J* = 6.9 Hz) 2.52–2.65 (m, 4H, piperazine) 2.74–2.88 (m, 1H, CH-pyrrolidyno-2,5-dione) 3.13–3.30 (m, 5H, piperazine, CH-pyrrolidyno-2,5-dione) 3.57–3.71 (m, 2H, CH_2_) 4.30 (dd, 1H, CH-pyrrolidyno-2,5-dione, *J* = 9.5, 5.2 Hz) 6.83 (d, 1H, thiophene, *J* = 5.1 Hz) 7.01–7.13 (m, 3H, ArH) 7.25 (d, 1H, thiophene, *J* = 1.5 Hz) 7.32 (t, 1H, ArH, *J* = 7.9 Hz); ^13^C NMR (126 MHz, CDCl_3_): δ ppm 14.21, 24.81, 29.12, 36.96, 39.81, 48.76, 52.99, 55.74, 112.17 (q, *J* = 3.82 Hz), 115.85 (q, *J* = 3.82 Hz), 118.73, 124.41 (q, *J* = 272.20 Hz), 123.06, 129.60, 130.83, 131.47 (q, *J* = 31.39 Hz), 132.37, 136.18, 151.44, 175.55, 176.88; LC/MS for base: C_23_H_26_ F_3_N_3_O_2_S (465.54); Monoisotopic Mass 465.17; [M + H]^+^ = 466.67.

##### (*R,S*)-1-(3-(4-(3-Chlorophenyl)piperazin-1-yl)propyl)-3-(3-methylthiophen-2-yl)pyrrolidine-2,5-dione hydrochloride (7)

White powdery crystals. Yield: 65%; mp. 181.5–183.5 °C; TLC: R_f_ = 0.49 (S_2_); UPLC: *t*_R_ = 4.95 min; ^1^H NMR (500 MHz, CDCl_3_): δ ppm 2.24 (s, 3H, CH_3_) 2.24–2.30 (m, 2H, CH_2_) 2.78 (dd, 1H, CH-pyrrolidyno-2,5-dione, *J* = 18.4, 5.2 Hz) 2.86–2.97 (m, 2H, piperazine) 3.07–3.14 (m, 2H, piperazine) 3.33 (dd, 1H CH-pyrrolidyno-2,5-dione, *J* = 18.4, 9.5 Hz) 3.59 (br. s., 6H, CH_2_, piperazine) 3.65–3.71 (m, 2H, CH_2_) 4.50 (dd, 1H, CH-pyrrolidyno-2,5-dione, *J* = 9.5, 5.1 Hz) 6.75 (ddd, 1H, thiophene, *J* = 8.3, 2.4, 0.7 Hz) 6.82 (d, 1H, ArH, *J* = 5.2 Hz) 6.86 (t, 1H, ArH *J* = 2.1 Hz) 6.89 (ddd, 1H, ArH, *J* = 7.9, 1.9, 0.7 Hz) 7.10 (d, 1H, thiophene, *J* = 5.2 Hz) 7.17 (t, 1H, ArH, *J* = 8.1 Hz); ^13^C NMR (126 MHz, CDCl_3_): δ ppm 14.25, 22.71, 37.80, 39.94, 46.34, 51.63, 54.79, 115.07, 117.29, 121.55, 123.06, 130.53, 130.92, 132.16, 135.29, 136.54, 150.51, 175.75, 177.26; LC/MS for base: C_22_H_26_ClN_3_O_2_S (431.98); Monoisotopic Mass 431.14; [M + H]^+^ = 432.18.

##### (*R,S*)-1-(3-(4-(4-Chlorophenyl)piperazin-1-yl)propyl)-3-(3-methylthiophen-2-yl)pyrrolidine-2,5-dione hydrochloride (8)

White powdery crystals. Yield: 60%; mp. 68.0–70.0 °C; TLC: R_f_ = 0.47 (S_2_); UPLC: *t*_R_ = 4.97 min; ^1^H NMR (500 MHz, CDCl_3_): δ ppm 1.71–1.82 (m, 2H, CH_2_) 2.19 (s, 3H, CH_3_) 2.37 (t, 2H, CH_2_, *J* = 7.05 Hz) 2.46–2.55 (m, 4H, piperazine) 2.69–2.77 (m, 1H, CH-pyrrolidyno-2,5-dione) 2.99–3.11 (m, 4H, piperazine) 3.17 (dd, 1H, CH-pyrrolidyno-2,5-dione, *J* = 18.4, 9.5 Hz) 3.59 (t, 2H, CH_2_, *J* = 7.16 Hz) 4.21–4.29 (m, 1H, CH-pyrrolidyno-2,5-dione), 6.72–6.81 (m, 3H, ArH, thiophene) 7.06 (d, 1H, thiophene, *J* = 5.16 Hz) 7.09–7.16 (m, 2H, ArH); ^13^C NMR (126 MHz, CDCl_3_): δ ppm 14.18, 24.76, 37.65, 39.51, 49.16, 53.00, 55.71, 117.19, 123.03, 124.39, 128.58, 130.78, 132.39, 136.11, 149.95, 175.53, 176.86; LC/MS for base: C_22_H_26_ClN_3_O_2_S (431.98); Monoisotopic Mass 431.14; [M + H]^+^ = 432.12.

##### (*R,S*)-1-(3-(4-(3,4-Dichlorophenyl)piperazin-1-yl)propyl)-3-(3-methylthiophen-2-yl)pyrrolidine-2,5-dione hydrochloride (9)

White powdery crystals. Yield: 55%; mp. 101.0–103.0 °C; TLC: R_f_ = 0.58 (S_1_); UPLC: *t*_R_ = 5.43 min; ^1^H NMR (500 MHz, CDCl_3_): δ ppm 2.22 (s, 3H, CH_3_) 2.23–2.28 (m, 2H, CH_2_) 2.76 (dd, 1H, CH-pyrrolidyno-2,5-dione, *J* = 18.4, 5.1 Hz) 2.73–2.73 (m, 2H, CH_2_) 3.09–3.16 (m, 4H, piperazine) 3.31 (dd, 1H, CH-pyrrolidyno-2,5-dione, *J* = 18.4, 9.6 Hz) 3.55 (d, 4H, piperazine, *J* = 4.9 Hz) 3.66 (t, 2H, CH_2_, *J* = 6.3 Hz) 4.50 (dd, 1H, CH-pyrrolidyno-2,5-dione, *J* = 9.5, 5.2 Hz) 6.71 (dd, 1H, thiophene, *J* = 8.9, 2.9 Hz) 6.81 (d, 1H, ArH, *J* = 5.2 Hz) 6.95 (d, 1H, ArH *J* = 2.9 Hz) 7.09 (d, 1H, thiophene, *J* = 5.1 Hz) 7.23–7.30 (m, 1H, ArH); ^13^C NMR (126 MHz, CDCl_3_): δ ppm 14.22, 22.68, 36.17, 37.79, 39.94, 46.35, 51.61, 54.76, 116.55, 118.82, 123.09, 124.61, 130.90, 132.13, 133.21, 136.50, 148.92, 175.85, 177.36; LC/MS for base: C_22_H_25_Cl_2_N_3_O_2_S (465.42); Monoisotopic Mass 465.10; [M + H]^+^ = 466.15.

##### General Procedure for the Synthesis of Compounds 10–17

The obtained 2-(3-(3-methylthiophen-2-yl)-2,5-dioxopyrrolidin-1-yl)acetic acid (**2**) (0.002 mol) was dissolved in 20 mL of DMF and N,N-carbonyldiimidazole (0.002 mol) was added. The mixtures were stirred for 0.5 h at a room temperature. Afterwards, the appropriate 4-arylpiperazine or morpholine (0.002 mol) was added. After 24 h of stirring the solvent was evaporated and the oily residue was purified by column chromatography (dichloromethane:methanol, 9:0.5, *v*/*v*) giving the final compounds **10**–**17** as racemic mixture in yields ranging from 31% to 59%.

##### (*R,S*)-3-(3-Methylthiophen-2-yl)-1-(2-morpholino-2-oxoethyl)pyrrolidine-2,5-dione (10)

White powdery crystals. Yield: 35.5%; mp. 142.0–144.0 °C; TLC: R_f_ = 0.59 (S_1_); UPLC: *t*_R_ = 4.21 min; ^1^H NMR (500 MHz, CDCl_3_): δ ppm 2.25 (s, 3H, CH_3_) 2.89 (dd, 1H, CH-pyrrolidyno-2,5-dione, *J* = 18.5, 5.7 Hz) 3.32 (dd, 1H, CH-pyrrolidyno-2,5-dione, *J* = 18.4, 9.6 Hz) 3.43–3.61 (m, 4H, morpholine) 3.65–3.73 (m, 4H, morpholine) 4.33 (d, 2H, CH_2_, *J* = 11.1 Hz) 4.45 (dd, 1H, CH-pyrrolidyno-2,5-dione, *J* = 9.7, 5.7 Hz)) 6.82 (d, 1H, thiophene, *J* = 5.2 Hz) 7.12 (d, 1H, thiophene, *J* = 5.2 Hz); ^13^C NMR (126 MHz, CDCl_3_): δ ppm 14.21, 37.73, 39.94, 42.56, 45.17, 65.71, 123.33, 130.73, 131.93, 136.39, 163.41, 175.09, 176.47; C_15_H_18_N_2_O_4_S (322.10); Monoisotopic Mass 322.38; [M + H]^+^ = 323.18.

##### (*R,S*)-1-(2-(4-(4-Fluorophenyl)piperazin-1-yl)-2-oxoethyl)-3-(3-methylthiophen-2-yl)pyrrolidine-2,5-dione (11)

White powdery crystals. Yield: 55.5%; mp. 165.0–166.0 °C; TLC: R_f_ = 0.67 (S_1_); UPLC: *t*_R_ = 6.33 min; ^1^H NMR (500 MHz, CDCl_3_): δ ppm 2.27 (s, 3H, CH_3_) 2.91 (dd, 1H, CH-pyrrolidyno-2,5-dione, *J* = 18.5, 5.7 Hz) 3.05–3.16 (m, 4H, piperazine) 3.35 (dd, 1H, CH-pyrrolidyno-2,5-dione, *J*=18.4, 9.6 Hz) 3.60–3.78 (m, 4H, piperazine) 4.40 (d, 2H, CH_2_, *J* = 10.7 Hz) 4.43–4.49 (m, 1H, CH-pyrrolidyno-2,5-dione) 6.83 (d, 1H, thiophene, *J* = 5.1 Hz) 6.86–6.90 (m, 2H, ArH) 6.94–7.01 (m, 2H, ArH) 7.13 (d, 1H, thiophene, *J* = 5.1 Hz); ^13^C NMR (126 MHz, CDCl_3_): δ ppm 14.22, 37.75, 39.99, 42.39, 44.88, 50.41, 115.87 (d, *J* = 22.33 Hz), 118.95 (d, *J* = 7.85 Hz), 130.74, 131.93, 136.41, 147.54 (d, *J* = 2.41 Hz), 157.87 (d, *J* = 240.23 Hz), 163.20, 175.12, 176.49; C_21_H_22_N_3_O_3_FS (415.48); Monoisotopic Mass 415.14; [M + H]^+^ = 416.10.

##### (*R,S*)-3-(3-Methylthiophen-2-yl)-1-(2-oxo-2-(4-(3-(trifluoromethyl)phenyl)piperazin-1-yl)ethyl)pyrrolidine-2,5-dione (12)

White powdery crystals. Yield: 55.2%; mp. 135.0–137.0 °C; TLC: R_f_ = 0.52 (S_1_); UPLC: *t*_R_ = 7.23 min; ^1^H NMR (500 MHz, CDCl_3_): δ ppm 2.27 (s, 3H, CH_3_) 2.92 (dd, *J* = 18.5, 5.7 Hz, 1H, CH-pyrrolidyno-2,5-dione, *J* = 18.5, 5.7 Hz) 3.19–3.31 (m, 4H, piperazine) 3.35 (dd, 1H, CH-pyrrolidyno-2,5-dione, *J* = 18.5, 9.7 Hz) 3.62–3.80 (m, 4H, piperazine) 4.40 (d, 2H, CH_2_, *J* = 10.5 Hz) 4.43–4.50 (m, 1H, CH-pyrrolidyno-2,5-dione) 6.83 (d, 1H, thiophene, *J* = 5.1 Hz) 7.05 (dd, 1H, thiophene, *J* = 8.3, 2.3 Hz) 7.09–7.16 (m, 3H, ArH) 7.37 (t, 1H, ArH, *J* = 7.9 Hz); ^13^C NMR (126 MHz, CDCl_3_): δ ppm 14.23, 37.27, 39.96, 41.52, 44.25, 48.34, 113.09 (q, *J* = 3.82 Hz), 117.07 (q, *J* = 4.02 Hz), 119.55, 124.23 (q, *J* = 272.20 Hz), 123.35, 129.88, 130.75, 131.71 (q, *J* = 31.99 Hz), 131.91, 136.42, 150.96, 163.32, 175.11, 176.48; C_22_H_22_N_3_O_3_F_3_S (465.49); Monoisotopic Mass 465.13; [M + H]^+^ = 466.08.

##### (*R,S*)-1-(2-(4-(2-Chlorophenyl)piperazin-1-yl)-2-oxoethyl)-3-(3-methylthiophen-2-yl)pyrrolidine-2,5-dione (13)

White powdery crystals. Yield: 31.0%; mp. 141.0–143.0 °C; TLC: R_f_ = 0.38 (S_1_); UPLC: *t*_R_ = 7.02 min; ^1^H NMR (500 MHz, CDCl_3_): δ ppm 2.27 (s, 3H, CH_3_) 2.92 (dd, 1H, CH-pyrrolidyno-2,5-dione, *J* = 18.4, 5.7 Hz) 3.03 (t, 2H, piperazine, *J* = 5.0 Hz) 3.07–3.13 (m, 2H, piperazine) 3.35 (dd, 1H, CH-pyrrolidyno-2,5-dione, *J* = 18.4, 9.7 Hz) 3.61–3.67 (m, 2H, piperazine) 3.76–3.81 (m, 2H, piperazine) 4.40 (d, 2H, CH_2_, *J* = 11.1 Hz) 4.43–4.50 (m, 1H, CH-pyrrolidyno-2,5-dione) 6.83 (d, 1H, thiophene, *J* = 5.2 Hz) 6.97–7.04 (m, 2H, ArH) 7.13 (d, *J* = 5.16 Hz, 1H, thiophene) 7.19–7.27 (m, 1H, ArH) 7.37 (dd, 1H, ArH, *J* = 8.3, 1.5 Hz); ^13^C NMR (126 MHz, CDCl_3_): δ ppm 14.22, 37.75, 40.04, 42.71, 45.19, 50.58, 120.71, 123.32, 124.56, 127.84, 129.08, 130.78, 131.98, 136.40, 148.54, 163.28, 175.14, 176.50; C_21_H_22_N_3_O_3_ClS (431.94); Monoisotopic Mass 431.11; [M + H]^+^ = 432.12.

##### (*R,S*)-1-(2-(4-(3-Chlorophenyl)piperazin-1-yl)-2-oxoethyl)-3-(3-methylthiophen-2-yl)pyrrolidine-2,5-dione (14)

White powdery crystals. Yield: 59.2%; mp. 117.0–119.0 °C; TLC: R_f_ = 0.76 (S_1_); UPLC: *t*_R_ = 6.99 min; ^1^H NMR (500 MHz, CDCl_3_): δ ppm 2.27 (s, 3H, CH_3_) 2.91 (dd, *J* = 18.5, 5.7 Hz, 1H, CH-pyrrolidyno-2,5-dione, *J* = 18.5, 5.7 Hz) 3.15–3.27 (m, 4H, piperazine) 3.34 (dd, 1H, CH-pyrrolidyno-2,5-dione, *J*=18.5, 9.7 Hz) 3.59–3.78 (m, 4H, piperazine) 4.39 (d, 2H, CH_2_, *J* = 10.7 Hz) 4.43–4.49 (m, 1H, CH-pyrrolidyno-2,5-dione) 6.74–6.81 (m, 1H, thiophene) 6.82–6.89 (m, 3H, ArH) 7.13 (d, 1H, thiophene, *J* = 5.15 Hz) 7.16–7.21 (m, 1H, ArH); ^13^C NMR (126 MHz, CDCl_3_): δ ppm 14.22, 37.75, 40.00, 42.13, 44.62, 48.94, 114.66, 116.69, 120.52, 123.34, 130.32, 130.74, 131.91, 135.18, 136.41, 151.85, 163.28, 175.09, 176.46; C_21_H_22_N_3_O_3_ClS (431.94); Monoisotopic Mass 431.11; [M + H]^+^ = 432.12.

##### (*R,S*)-1-(2-(4-(4-Chlorophenyl)piperazin-1-yl)-2-oxoethyl)-3-(3-methylthiophen-2-yl)pyrrolidine-2,5-dione (15)

White powdery crystals. Yield: 55.5%; mp. 179.0–181.0 °C; TLC: R_f_ = 0.71 (S_1_); UPLC: *t*_R_ = 6.93 min; ^1^H NMR (500 MHz, CDCl_3_): δ ppm 2.26 (s, 3H, CH_3_) 2.91 (dd, 1H, CH-pyrrolidyno-2,5-dione, *J* = 18.5, 5.7 Hz) 3.10–3.23 (m, 4H, piperazine) 3.34 (dd, *J*=18.4, 9.7 Hz, 1H, CH-pyrrolidyno-2,5-dione) 3.59–3.78 (m, 4H, piperazine) 4.39 (d, *J* = 10.7 Hz, 2H, CH_2_) 4.42–4.49 (m, 1H, CH-pyrrolidyno-2,5-dione) 6.80–6.86 (m, 3H, ArH, thiophene) 7.13 (d, 1H, thiophene, *J* = 5.2 Hz) 7.19–7.24 (m, 2H, ArH); ^13^C NMR (126 MHz, CDCl_3_): δ ppm 14.22, 37.75, 40.00, 42.19, 44.69, 48.93, 118.15, 123.34, 125.82, 128.59, 130.75, 131.93, 136.41, 149.47, 163.24, 175.11, 176.48; C_21_H_22_N_3_O_3_ClS (431.94); Monoisotopic Mass 431.11; [M + H]^+^ = 432.12.

##### (*R,S*)-1-(2-(4-(2,3-Dichlorophenyl)piperazin-1-yl)-2-oxoethyl)-3-(3-methylthiophen-2-yl)pyrrolidine-2,5-dione (16)

White powdery crystals. Yield: 62%; mp. 112.0–113.5 °C; TLC: R_f_ = 0.71 (S_1_); UPLC: *t*_R_ = 7.52 min; ^1^H NMR (500 MHz, CDCl_3_): δ ppm 2.27 (s, 3H, CH_3_) 2.91 (dd, 1H, CH-pyrrolidyno-2,5-dione, *J* = 18.4, 5.7 Hz) 3.01 (t, 2H, piperazine, *J* = 4.9 Hz) 3.06–3.11 (m, 2H, piperazine) 3.34 (dd, 1H, CH-pyrrolidyno-2,5-dione, *J* = 18.4, 9.7 Hz) 3.61–3.68 (m, 2H, piperazine) 3.78 (br. s., 2H, piperazine) 4.40 (d, 2H, CH_2_, *J* = 11.1 Hz) 4.43–4.50 (m, 1H, CH-pyrrolidyno-2,5-dione) 6.83 (d, 1H, thiophene, *J* = 5.2 Hz) 6.92 (dd, 1H, ArH, *J* = 7.9, 1.7 Hz) 7.13 (d, 1H, thiophene, *J* = 5.2 Hz) 7.16 (d, 1H, ArH, *J* = 7.9 Hz) 7.18-7.21 (m, 1H, ArH); ^13^C NMR (126 MHz, CDCl_3_): δ ppm 14.22, 37.75, 40.02, 42.65, 45.15, 50.40, 118.95, 123.33, 125.50, 127.41, 130.74, 131.95, 134.31, 136.40, 150.47, 163.33, 175.13, 176.50; C_21_H_21_N_3_O_3_Cl_2_S (466.38); Monoisotopic Mass 465.08; [M + H]^+^ = 466.28.

##### (*R,S*)-1-(2-(4-(3,4-Dichlorophenyl)piperazin-1-yl)-2-oxoethyl)-3-(3-methylthiophen-2-yl)pyrrolidine-2,5-dione (17)

White powdery crystals. Yield: 49.5%; mp. 141.0–143.0 °C; TLC: R_f_ = 0.74 (S_1_); UPLC: *t*_R_ = 7.48 min; ^1^H NMR (500 MHz, CDCl_3_): δ ppm 2.27 (s, 3H, CH_3_) 2.89–2.96 (m, 1H CH-pyrrolidyno-2,5-dione) 3.15 (t, 2H, piperazine, *J* = 5.1 Hz) 3.19–3.25 (m, 2H, piperazine) 3.35 (dd, 1H, CH-pyrrolidyno-2,5-dione, *J* = 18.5, 9.6 Hz) 3.60–3.66 (m, 2H, piperazine) 3.72–3.78 (m, 2H, piperazine) 4.39 (d, 2H, CH_2_, *J* = 10.5 Hz) 4.42–4.49 (m, 1H CH-pyrrolidyno-2,5-dione) 6.73 (dd, 1H, thiophene, *J* = 8.9, 2.9 Hz) 6.96 (d, 1H, ArH, *J* = 2.9 Hz) 7.13 (d, 1H, thiophene, *J* = 5.2 Hz) 7.25 (s, 1H, ArH) 7.29 (d, 1H, ArH, *J* = 8.9 Hz); ^13^C NMR (126 MHz, CDCl_3_): δ ppm 14.22, 37.75, 39.98, 42.03, 44.53, 48.92, 116.09, 118.18, 122.97, 130.75, 131.88, 133.11, 136.42, 150.18, 163.30, 175.08, 176.46; C_21_H_21_N_3_O_3_Cl_2_S (466.38); Monoisotopic Mass 465.08; [M + H]^+^ = 466.28.

### 4.2. In Vivo Experiments

#### 4.2.1. Animals

Experiments were carried out using adult male CD-1 mice weighing 20–26 g, purchased from the Animal House at the Faculty of Pharmacy, Jagiellonian University Medical College, Krakow, Poland. The animals were housed in groups of 10 in the standard plastic cages, at room temperature of 20 ± 2 °C, exposed to 12:12 h light/dark cycle, with ad libitum standard laboratory food and water. All experiments were performed between 9 a.m. and 3 p.m. The tested groups consisting of 4–8 mice were chosen by means of a randomized schedule. Behavioral measures were scored by trained observers. All procedures performed in studies involving animals were in accordance with the ethical standards of the institution or practice at which the studies were conducted, and were approved by the Local Ethical Committee in Kraków, Poland (Approval No: 131/2017, 159/2018, 287B/2019, 288/2019, 209A/2020).

#### 4.2.2. The Maximal Electroshock Seizure Test (MES Test)

The MES test was performed as previously described [46]. Stimuli (25 mA, 0.2 s, 50 Hz) were generated by a constant current stimulator (Rodent shocker, Type 221, Hugo Sachs Elektronik, Germany) and delivered with the use of auricular electrodes. During the stimulation, each mouse was restrained manually, and immediately after the stimulation gently released for observation. The endpoint was the tonic extension of the hind limbs. Mice not displaying hindlimb tonic extension were considered to be protected from seizure. The initial anticonvulsant screening of the tested compounds was performed at a fixed dose of 100 mg/kg (four mice per group). The ED_50_ was calculated for active compounds and defined as the dose of a drug protecting 50% of animals against seizures and calculated according to the log-probit method [47]. To evaluate the ED_50_, at least three groups of animals were injected with various doses of tested compounds. Each group consisted of six animals.

#### 4.2.3. The 6 Hz Psychomotor Seizure Test

Psychomotor seizures were induced via corneal stimulation (6 Hz, 32 mA, 0.2 ms rectangular pulse width, 3 s duration) using a constant-current device (ECT Unit 57800, Ugo Basile, Italy). A drop of 1% solution of lidocaine hydrochloride was applied to the mouse corneas before stimulation to provide local anesthesia and ensure optimal current conductivity. After the electrical stimulation, mice were gently released and observed for the presence or absence of seizure activity, being characterized by immobility associated with rearing, forelimb clonus, twitching of the vibrissae, stun, and Straub-tail. Lack of the above symptoms or their cessation within 10 s from the stimulation was considered as absence of seizure activity [48]. The initial anticonvulsant screening of the tested compounds was performed at a fixed dose of 100 mg/kg (four mice per group). Then ED_50_ was calculated for active compounds as described above.

#### 4.2.4. PTZ Seizure Test

Clonic convulsions were induced by the subcutaneous (*sc*) administration of PTZ at a dose of 100 mg/kg. After PTZ injection, the mice were placed separately and observed during the next 30 min for the occurrence of clonic seizures. Clonic seizures were defined as clonus of the whole body lasting more than 3 s, with an accompanying loss of righting reflex. The latency to first clonus, the number of seizure episodes, and mortality rate were noted and compared with vehicle-treated and drug-treated groups. The absence of clonic convulsions within the observed time period was interpreted as the compound’s ability to protect against PTZ-induced seizures [14].

#### 4.2.5. Rotarod Test

In this test mice were trained to balance on an accelerating rotarod that rotated at 10 rotations per minute (Rotarod apparatus, May Commat RR0711, Turkey; rod diameter: 3 cm). During the training session, the animals were placed on a rotating rod for 3 min with an unlimited number of trials. Proper experiment was conducted 24 h after the training trial. On the test day, trained mice were intraperitoneally pretreated with the test compound at a dose of 100 mg/kg and just before the MES test were tested on the rotarod. Neurotoxicity was indicated by the inability of the animal to maintain equilibration on the rod for 1 min. To evaluate the TD_50_ value animals were injected at a dose 200 or 300 mg/kg of the test compound. Each group consisted of six animals [12].

#### 4.2.6. Writhing Test

Mice were injected with the test compound or vehicle by intraperitoneally route (*ip*), and 30 min later, 0.9 % acetic acid solution was injected *ip* and the animals were placed into the glass beakers. The writhing response (lengthwise stretching of the torso, concave arching of the back, and extension of the hind limbs) was recorded for 30 min. The analgesic effect of the tested substances was determined by a decrease in the number of writhes observed and the results were expressed as percent inhibition of writhing behavior (as index of analgesia), and was calculated using the following formula:

%Inhibition=(NA−NB)/NA×100,

where NA—number of writhes in vehicle group, NB—number of writhes in drug-treated group.

#### 4.2.7. Hot Plate Test

The hot plate test was carried out based on the Eddy and Leimbach (1953) method. In this test, the apparatus was equipped with an electrically heated surface with a temperature controller that maintains surface temperature to a set point. Herein, the temperature was set at 55 °C. The animals were treated *ip* either with the test compounds, or vehicle 30 min before placing the animal on a hot plate apparatus. Latency time to pain reaction, i.e., the time until the animal licked its back paws or jumped was recorded by means of the stopwatch. Mice that did not respond to elevated temperature during 60 s were removed from the apparatus.

### 4.3. In Vitro Experiments

#### 4.3.1. Binding and Functional Assays

Binding and functional studies were performed commercially in Cerep Laboratories (Poitiers, France) using testing procedures described elsewhere (Na^+^ channel-site 2 [27], L-type Ca^2+^ channel, dihydropyridine site [28], TRPV_1_ (VR_1_) (h) (antagonist effect) [49], GABA transporter (GAT) [29]). All experiments were performed in duplicate.

#### 4.3.2. Analysis of Hepatotoxicity using Cellular Model

Hepatotoxicity of tested compounds was assessed in PrestoBlue (Thermo Fisher Scientific, Waltham, MA, USA) Experiments on microplate reader (SpectraMax^®^ iD3, Molecular Devices, San Jose, CA, USA) using human hepatocellular carcinoma cells (Hep G2) ATCC^®^ 59195™. The cells were cultured in standard conditions (37 °C, 5% CO_2_), in Dulbecco’s Modified Eagle’s Medium-high glucose, (DMEM, Gibco, Thermo Fisher Scientific, Waltham, MA, USA) supplemented with 10% fetal bovine serum (Thermo Fisher Scientific, Waltham, MA, USA), with added 100 IU/mL penicillin (Sigma Aldrich, Steinheim, Germany) and 100 µg/mL streptomycin (Sigma Aldrich). PrestoBlue reagent is a resazurin-based solution that functions as a cell viability indicator. Metabolically active cells are capable of reducing the PrestoBlue reagent, with the colorimetric changes used as an indicator to quantify the viability of cells in culture. This change can be determined by measuring the fluorescence. After 24 h of incubation with the compounds PrestoBlue reagent was added to wells of a microplate. After 20 min of incubation at 37 °C, the fluorescence intensity (EX 530; EM 580 nm) was measured in a plate reader. Viability values were calculated as a percentage of live cells with respect to the control sample (DMSO). The negative control was medium without cells. Astemizole was used as a reference substance [50].

## 5. Conclusions

A library of 15 new compounds with good drug likeness properties was synthesized and evaluated for anticonvulsant and neurotoxic properties in mice. The obtained results revealed that part of compounds exhibited anticonvulsant activity, especially in the psychomotor seizure (6 Hz) test, which is the animal model of partial type of seizures and pharmacoresistant epilepsy. In addition, three molecules, namely **3**, **4**, and **6** exhibited beneficial ED_50_ values as compared to valproic acid and ethosuximide in the 6 Hz test. The highest activity was observed for 3-(3-methylthiophen-2-yl)-1-(3-morpholinopropyl)-pyrrolidine-2,5-dione hydrochloride (**4**) that showed ED_50_ value of 62.14 mg/kg in the MES test and 75.59 mg/kg in the 6 Hz test. Furthermore, in vitro studies showed that blockage of the neuronal voltage-sensitive sodium (site-2) and L-type calcium channels could be a possible mechanism of action of compounds **4** and **6**. None of the tested compounds showed interaction with the GABA transporter. Additionally, four of the tested compounds (**3**, **4**, **6**, and **9**) revealed peripheral analgesic activity in the writhing test. The strongest effect was observed for compound **9** at the dose of 30 mg/kg, and it was similar to that of aspirin at the same dose. Two compounds (**6** and **9**) at the dose of 30 mg/kg revealed central analgesic activity in the hot plate test. Moreover, compounds **6** and **9** showed high affinity for TRPV1 receptors, which may imply their involvement in analgesic mechanisms.

## Figures and Tables

**Table 1:** Drug-likeness parameters according to Lipinski and Veber rules.

Comps	Lipinski Rules					Veber Rules	
	MW ≤ 500	LogP ≤ 5	NHD ≤ 5 ^a^	NHA ≤ 10 ^b^	Violations of Rules	NBR ≤ 10 ^c^	TPSA ≤ 140 ^d^
**3**	308.40	2.81	0	4	0	4	78.09
**4**	322.42	3.01	0	4	0	5	78.09
**5**	415.52	3.66	0	4	0	6	72.10
**6**	465.53	3.77	0	6	0	7	72.10
**7**	431.98	3.65	0	3	0	6	72.10
**8**	431.98	3.82	0	3	0	6	72.10
**9**	466.62	3.94	0	3	0	6	72.10
**10**	318.43	2.99	0	2	0	4	61.02
**11**	415.48	3.19	0	4	0	5	89.17
**12**	465.49	3.16	0	6	0	6	89.17
**13**	431.94	3.12	0	3	0	5	89.17
**14**	431.94	3.52	0	3	0	5	89.17
**15**	431.94	3.33	0	3	0	5	89.17
**16**	466.38	3.34	0	3	0	5	89.17
**17**	466.38	3.53	0	3	0	5	89.17

^a^ NHD: number of hydrogen bond donors; ^b^ NHA: number of hydrogen bond acceptors; ^c^ NBR: number of rotatable bonds; ^d^ TPSA: total polar surface area.

**Table 2:**
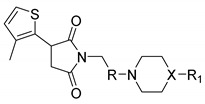
Anticonvulsant activity (MES, 6 Hz, *sc*PTZ) and acute neurotoxicity (rotarod test) following 0.5 h after *ip* administration in mice dose of 100 mg/kg.

Compd	R	X/R_1_	Intraperitoneal Administration in Mice		
			MES ^a^	6 Hz ^b^	NT ^c^
**3**	-CH_2_-	O/-	**1/4**	**4/4**	0/4
**4**	-(CH_2_)_2_-	O/-	**3/4**	**3/4**	0/4
**5**	-(CH_2_)_2_-	N/4-FPh	0/4	0/4	**4/4**
**6**	-(CH_2_)_2_-	N/3-CF_3_Ph	**1/4**	**3/4**	**4/4**
**7**	-(CH_2_)_2_-	N/3-ClPh	0/4	0/4	**2/4**
**8**	-(CH_2_)_2_-	N/4-ClPh	**1/4**	**1/4**	0/4
**9**	-(CH_2_)_2_-	N/3,4-ClPh	**2/4**	**2/4**	0/4
**10**	-(CO)-	O/-	0/4	**1/4**	0/4
**11**	-(CO)-	N/4-FPh	0/4	0/4	0/4
**12**	-(CO)-	N/3-CF_3_Ph	0/4	0/4	1/4
**13**	-(CO)-	N/2-ClPh	0/4	**1/4**	0/4
**14**	-(CO)-	N/3-ClPh	0/4	**2/4**	0/4
**15**	-(CO)-	N/4-ClPh	0/4	**1/4**	0/4
**16**	-(CO)-	N/2,3-ClPh	0/4	0/4	0/4
**17**	-(CO)-	N/3,4-ClPh	0/4	**2/4**	0/4

Ratios where at least one animal was protected or with motor impairment are highlighted in bold for easier data interpretation. Data indicate number of mice protected or with motor impairment/number of mice tested. The animals were examined at 0.5 h after administration of compounds at dose of 100 mg/kg. ^a^ MES—maximal electroshock seizures test. ^b^ 6 Hz (32 mA) —psychomotor seizure test. ^c^ NT—neurotoxicity screening—rotarod test.

**Table 3:** Quantitative pharmacological parameters ED_50_, TD_50_, and PI in mice *ip.*

Compd	TPE ^a^	MES ED_50_^b^ (mg/kg)	6 Hz ED_50 _^b^ (mg/kg)	NT TD_50_^b^ (mg/kg)	PI ^c^
**3**	0.5	-	74.32 (64.91–85.09)	> 200	> 2.69
**4**	0.5	62.14 (43.30–89.20)	75.59 (65.39–87.39)	< 200*	< 3.21 (MES) < 2.65 (6 Hz)
**6**	0.5	-	78.30 (65.82–93.13)	< 100	< 1.28
**9**	0.5	> 130	> 130	> 300	-
**14**	0.5	-	153.25 (130.70–179.68)	> 300	> 1.95 (6 Hz)
**17**	0.5	-	> 150	-	-
**ETX ^d^**	0.25	> 500	221.7 (183.0–268.5)	722.1 (647.0–805.8)	3.2 (6 Hz)
**VPA ^d^**	0.5	252.7 (220.1–290.2)	130.6 (117.6–145.2)	430.8 (407.9–454.9)	1.7 (MES) 3.3 (6 Hz)

Results are represented as mean ± SEM at 95% confidence limit. A dash indicates—not tested. ^a^ Time to peak effect. ^b^ MES—maximal electroshock test; 6 Hz—psychomotor seizure test (32 mA); NT—neurotoxicity—rotarod screen. ^c^ Protective index (TD_50_/ED_50_). ^d^ Reference AEDs: ETX—ethosuximide and VPA—valproic acid; tested in the same condition. Data from literature [25,26]. * Rapid death of animals after compounds administration at dose of 200 mg/kg.

**Table 4:** In vitro binding/functional assays (concentration 100 µM).

Compd	% Inhibition of Control Specific Binding ^a^		
	Voltage-Sensitive Na^+^ Channel (site 2) ^b^	Voltage-Sensitive Ca^2+^ Channel L-typ ^c^	GABA Transporter (GAT) ^d^
**3**	15.6%	−1.1%	−1.1%
**4**	42.1%	39.7%	−3.2%
**6**	96.7%	69.9%	-
**9**	78.8%	−9.5%	-

^a^ Results showing an inhibition higher than 50% are considered to represent significant effects of the tested compounds; results showing an inhibition between 25% and 50% are indicative of moderate effect; results showing an inhibition lower than 25% are not considered significant and mostly attributable to variability of the signal around the control level. ^b^ Compound was evaluated in synaptoneurosomal preparations from rat cerebral cortex as inhibitors of the specific binding of [^3^H] BTX to the voltage-sensitive sodium [27]. ^c^ Compound was evaluated in synaptoneurosomal preparations from rat cerebral cortex as inhibitors of the specific binding of [^3^H] nitrendipine to the voltage-sensitive Ca^2+^ channel [28]. ^d^ Compound was evaluated in synaptoneurosomal preparations from rat cerebral cortex as inhibitors of the specific binding of [^3^H] GABA (+ 10 µM isoguvacine) (+ 10 µM baclofen) to the GABA transporter [29].
